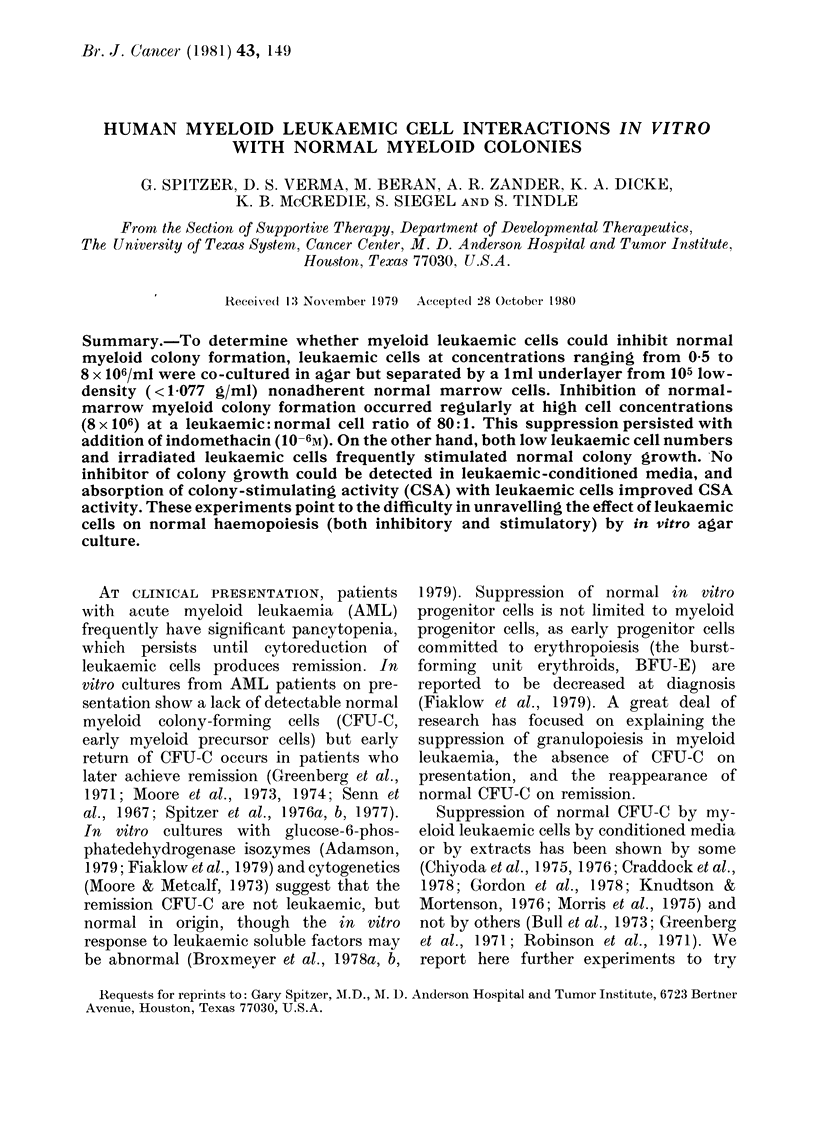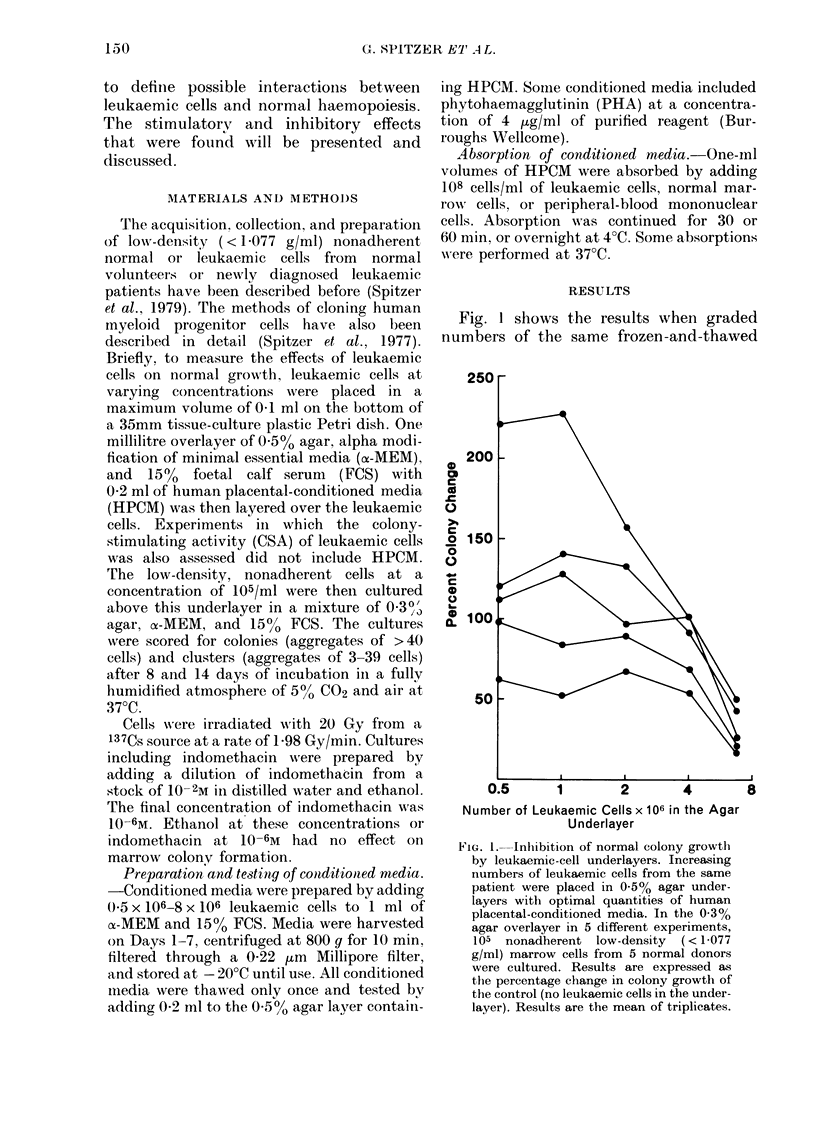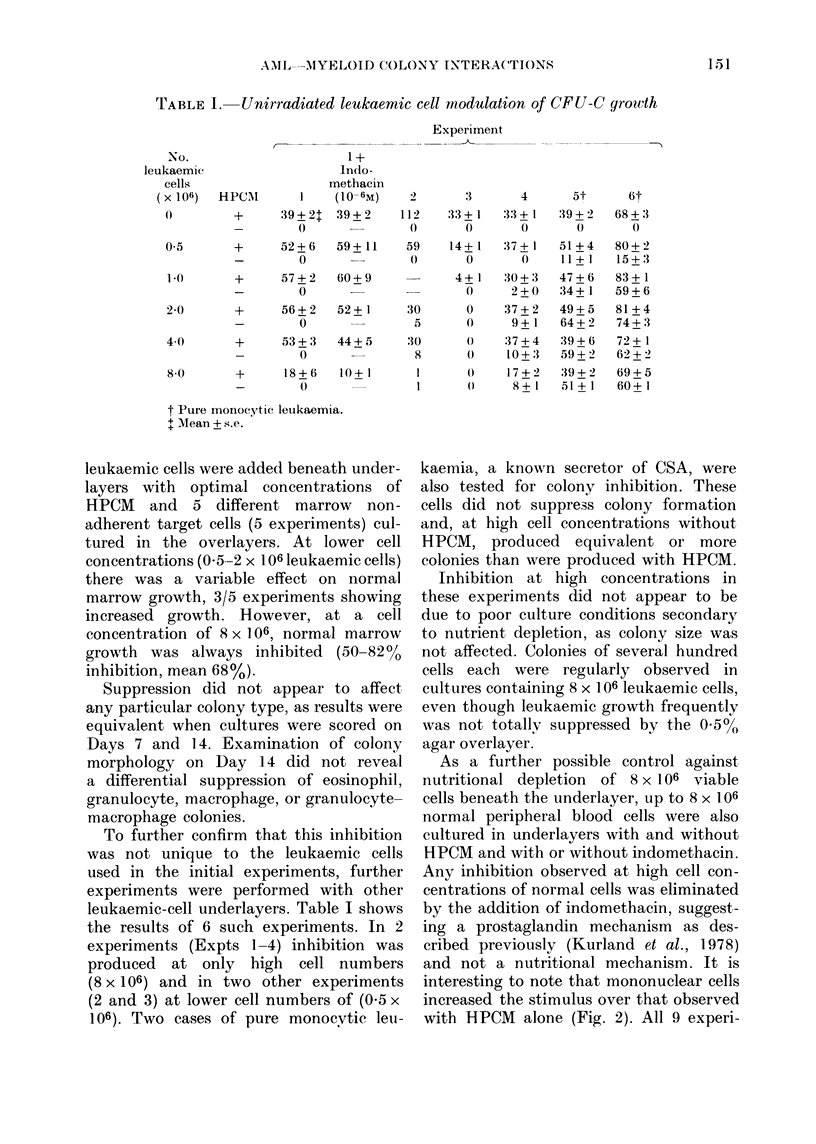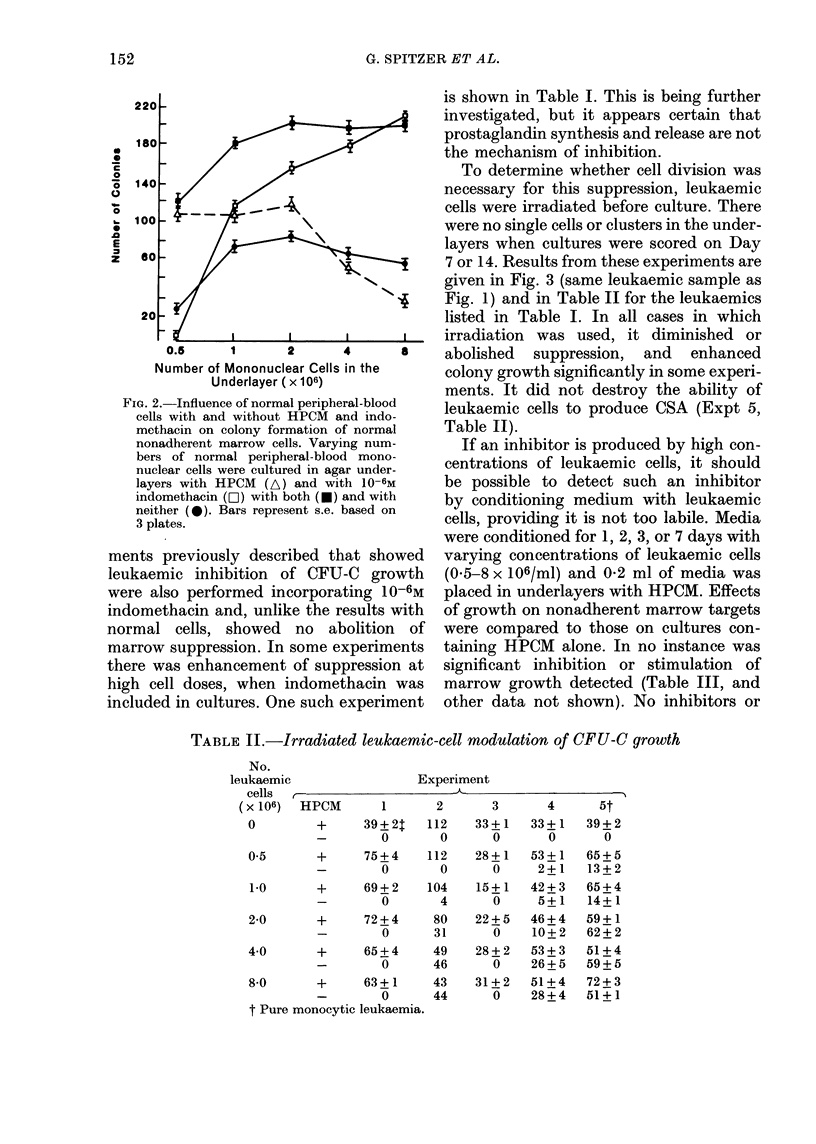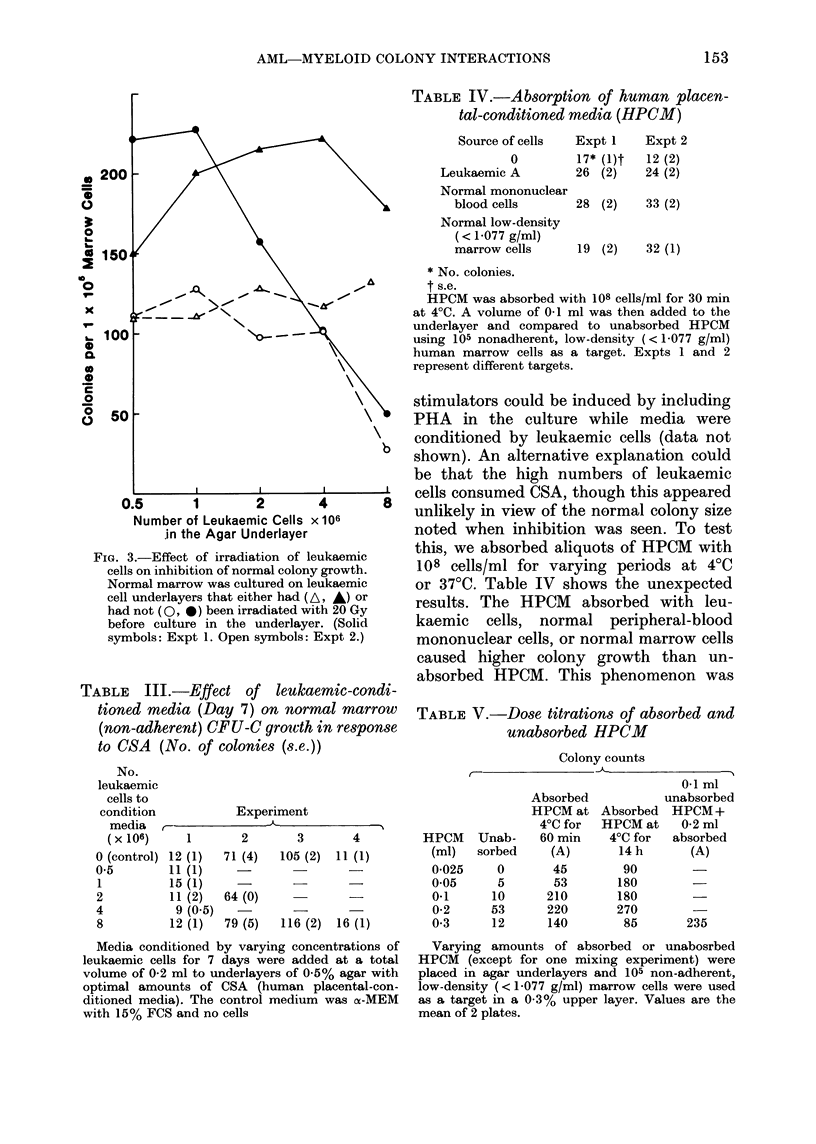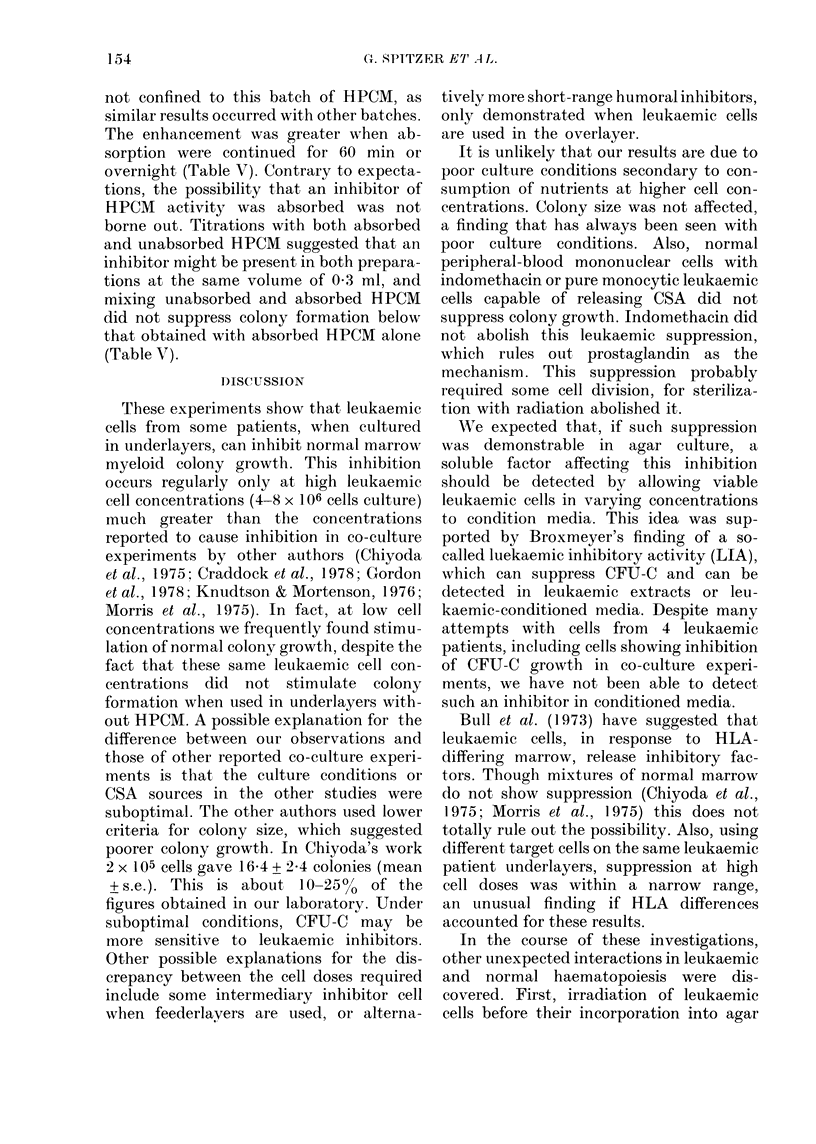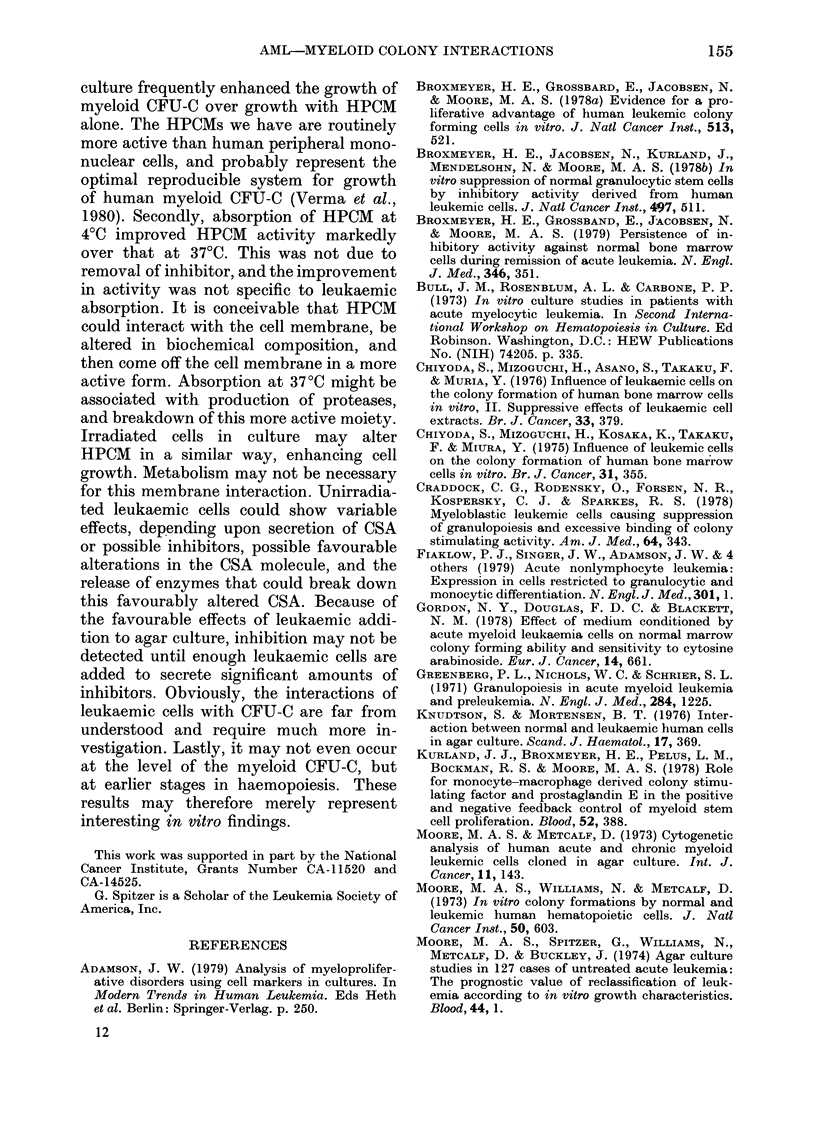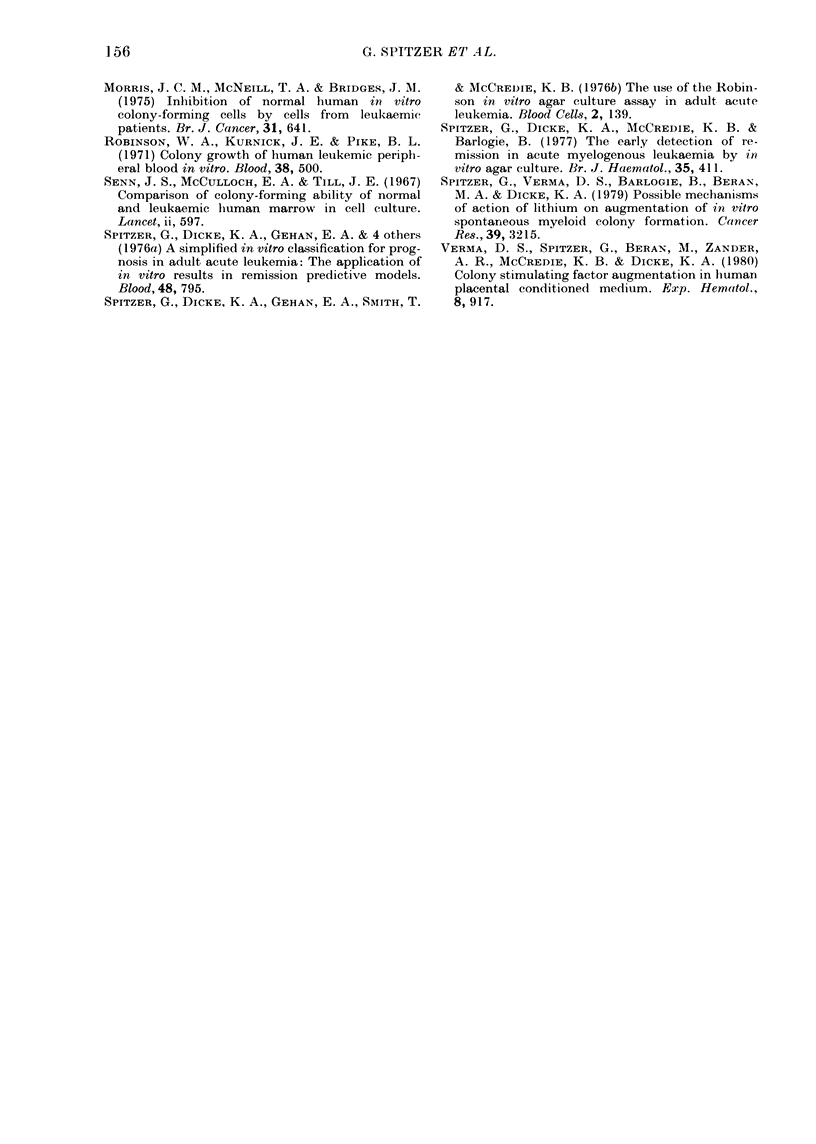# Human myeloid leukaemic cell interactions in vitro with normal myeloid colonies.

**DOI:** 10.1038/bjc.1981.23

**Published:** 1981-02

**Authors:** G. Spitzer, D. S. Verma, M. Beran, A. R. Zander, K. A. Dicke, K. B. McCredie, S. Siegel, S. Tindle

## Abstract

To determine whether myeloid leukaemic cells could inhibit normal myeloid colony formation, leukaemic cells at concentrations ranging from 0.5 to 8 X 10(6)/ml were co-cultured in agar but separated by a 1 ml underlayer from 10(5) low-density (less than 1.077 g/ml) nonadherent normal marrow cells. Inhibition of normal-marrow myeloid colony formation occurred regularly at high cell concentrations (8 X 10(6)) at a leukaemic:normal cell ratio of 80:1. This suppression persisted with addition of indomethacin (10(-6)M). On the other hand, both low leukaemic cell numbers and irradiated leukaemic cells frequently stimulated normal colony growth. No inhibitor of colony growth could be detected in leukaemic-conditioned media, and absorption of colony-stimulating activity (CSA) with leukaemic cells improved CSA activity. These experiments point to the difficulty in unravelling the effect of leukaemic cells on normal haemopoiesis (both inhibitory and stimulatory) by in vitro agar culture.


*Br. J. Cancer* (1981) **43**, 149

### HUMAN MYELOID LEUKAEMIC CELL INTERACTIONS *IN VITRO* WITH NORMAL MYELOID COLONIES

G. SPITZER, D. S. VERMA, M. BERAN, A. R. ZANDER, K. A. DICKE, K. B. McCREDIE, S. SIEGEL AND S. TINDLE

*From the Section of Supportive Therapy, Department of Developmental Therapeutics, The University of Texas System, Cancer Center, M. D. Anderson Hospital and Tumor Institute, Houston, Texas* 77030, *U.S.A.*

Received 13 November 1979 Accepted 28 October 1980

**Summary.—To determine whether myeloid leukaemic cells could inhibit normal myeloid colony formation, leukaemic cells at concentrations ranging from 0·5 to $8 \times 10^6$/ml were co-cultured in agar but separated by a 1ml underlayer from $10^5$ low-density (<1·077 g/ml) nonadherent normal marrow cells. Inhibition of normal-marrow myeloid colony formation occurred regularly at high cell concentrations ($8 \times 10^6$) at a leukaemic:normal cell ratio of 80:1. This suppression persisted with addition of indomethacin ($10^{-6}$M). On the other hand, both low leukaemic cell numbers and irradiated leukaemic cells frequently stimulated normal colony growth. No inhibitor of colony growth could be detected in leukaemic-conditioned media, and absorption of colony-stimulating activity (CSA) with leukaemic cells improved CSA activity. These experiments point to the difficulty in unravelling the effect of leukaemic cells on normal haemopoiesis (both inhibitory and stimulatory) by *in vitro* agar culture.**

At CLINICAL PRESENTATION, patients with acute myeloid leukaemia (AML) frequently have significant pancytopenia, which persists until cytoreduction of leukaemic cells produces remission. *In vitro* cultures from AML patients on presentation show a lack of detectable normal myeloid colony-forming cells (CFU-C, early myeloid precursor cells) but early return of CFU-C occurs in patients who later achieve remission (Greenberg *et al.*, 1971; Moore *et al.*, 1973, 1974; Senn *et al.*, 1967; Spitzer *et al.*, 1976*a*, *b*, 1977). *In vitro* cultures with glucose-6-phosphatedehydrogenase isozymes (Adamson, 1979; Fiaklow *et al.*, 1979) and cytogenetics (Moore & Metcalf, 1973) suggest that the remission CFU-C are not leukaemic, but normal in origin, though the *in vitro* response to leukaemic soluble factors may be abnormal (Broxmeyer *et al.*, 1978*a*, *b*, 1979). Suppression of normal *in vitro* progenitor cells is not limited to myeloid progenitor cells, as early progenitor cells committed to erythropoiesis (the burst-forming unit erythroids, BFU-E) are reported to be decreased at diagnosis (Fiaklow *et al.*, 1979). A great deal of research has focused on explaining the suppression of granulopoiesis in myeloid leukaemia, the absence of CFU-C on presentation, and the reappearance of normal CFU-C on remission.

Suppression of normal CFU-C by myeloid leukaemic cells by conditioned media or by extracts has been shown by some (Chiyoda *et al.*, 1975, 1976; Craddock *et al.*, 1978; Gordon *et al.*, 1978; Knudtson & Mortenson, 1976; Morris *et al.*, 1975) and not by others (Bull *et al.*, 1973; Greenberg *et al.*, 1971; Robinson *et al.*, 1971). We report here further experiments to try

Requests for reprints to: Gary Spitzer, M.D., M. D. Anderson Hospital and Tumor Institute, 6723 Bertner Avenue, Houston, Texas 77030, U.S.A.

to define possible interactions between leukaemic cells and normal haemopoiesis. The stimulatory and inhibitory effects that were found will be presented and discussed.

### MATERIALS AND METHODS

The acquisition, collection, and preparation of low-density (< 1·077 g/ml) nonadherent normal or leukaemic cells from normal volunteers or newly diagnosed leukaemic patients have been described before (Spitzer *et al.*, 1979). The methods of cloning human myeloid progenitor cells have also been described in detail (Spitzer *et al.*, 1977). Briefly, to measure the effects of leukaemic cells on normal growth, leukaemic cells at varying concentrations were placed in a maximum volume of 0·1 ml on the bottom of a 35mm tissue-culture plastic Petri dish. One millilitre overlayer of 0·5% agar, alpha modification of minimal essential media (α-MEM), and 15% foetal calf serum (FCS) with 0·2 ml of human placental-conditioned media (HPCM) was then layered over the leukaemic cells. Experiments in which the colony-stimulating activity (CSA) of leukaemic cells was also assessed did not include HPCM. The low-density, nonadherent cells at a concentration of $10^5$/ml were then cultured above this underlayer in a mixture of 0·3% agar, α-MEM, and 15% FCS. The cultures were scored for colonies (aggregates of > 40 cells) and clusters (aggregates of 3–39 cells) after 8 and 14 days of incubation in a fully humidified atmosphere of 5% $CO_2$ and air at 37°C.

Cells were irradiated with 20 Gy from a $^{137}Cs$ source at a rate of 1·98 Gy/min. Cultures including indomethacin were prepared by adding a dilution of indomethacin from a stock of $10^{-2}$M in distilled water and ethanol. The final concentration of indomethacin was $10^{-6}$M. Ethanol at these concentrations or indomethacin at $10^{-6}$M had no effect on marrow colony formation.

*Preparation and testing of conditioned media.*—Conditioned media were prepared by adding $0{\cdot}5 \times 10^6$–$8 \times 10^6$ leukaemic cells to 1 ml of α-MEM and 15% FCS. Media were harvested on Days 1–7, centrifuged at 800 *g* for 10 min, filtered through a 0·22 μm Millipore filter, and stored at −20°C until use. All conditioned media were thawed only once and tested by adding 0·2 ml to the 0·5% agar layer containing HPCM. Some conditioned media included phytohaemagglutinin (PHA) at a concentration of 4 μg/ml of purified reagent (Burroughs Wellcome).

*Absorption of conditioned media.*—One-ml volumes of HPCM were absorbed by adding $10^8$ cells/ml of leukaemic cells, normal marrow cells, or peripheral-blood mononuclear cells. Absorption was continued for 30 or 60 min, or overnight at 4°C. Some absorptions were performed at 37°C.

### RESULTS

Fig. 1 shows the results when graded numbers of the same frozen-and-thawed

FIG. 1.—Inhibition of normal colony growth by leukaemic-cell underlayers. Increasing numbers of leukaemic cells from the same patient were placed in 0·5% agar underlayers with optimal quantities of human placental-conditioned media. In the 0·3% agar overlayer in 5 different experiments, $10^5$ nonadherent low-density (< 1·077 g/ml) marrow cells from 5 normal donors were cultured. Results are expressed as the percentage change in colony growth of the control (no leukaemic cells in the underlayer). Results are the mean of triplicates.

TABLE I.—*Unirradiated leukaemic cell modulation of CFU-C growth*

| No. leukaemic cells (× 10⁶) | HPCM | Experiment 1 | 1 + Indomethacin ($10^{-6}$M) | 2 | 3 | 4 | 5† | 6† |
|---|---|---|---|---|---|---|---|---|
| 0 | + | 39 ± 2‡ | 39 ± 2 | 112 | 33 ± 1 | 33 ± 1 | 39 ± 2 | 68 ± 3 |
| | − | 0 | — | 0 | 0 | 0 | 0 | 0 |
| 0·5 | + | 52 ± 6 | 59 ± 11 | 59 | 14 ± 1 | 37 ± 1 | 51 ± 4 | 80 ± 2 |
| | − | 0 | — | 0 | 0 | 0 | 11 ± 1 | 15 ± 3 |
| 1·0 | + | 57 ± 2 | 60 ± 9 | — | 4 ± 1 | 30 ± 3 | 47 ± 6 | 83 ± 1 |
| | − | 0 | — | — | 0 | 2 ± 0 | 34 ± 1 | 59 ± 6 |
| 2·0 | + | 56 ± 2 | 52 ± 1 | 30 | 0 | 37 ± 2 | 49 ± 5 | 81 ± 4 |
| | − | 0 | — | 5 | 0 | 9 ± 1 | 64 ± 2 | 74 ± 3 |
| 4·0 | + | 53 ± 3 | 44 ± 5 | 30 | 0 | 37 ± 4 | 39 ± 6 | 72 ± 1 |
| | − | 0 | — | 8 | 0 | 10 ± 3 | 59 ± 2 | 62 ± 2 |
| 8·0 | + | 18 ± 6 | 10 ± 1 | 1 | 0 | 17 ± 2 | 39 ± 2 | 69 ± 5 |
| | − | 0 | — | 1 | 0 | 8 ± 1 | 51 ± 1 | 60 ± 1 |

† Pure monocytic leukaemia.

‡ Mean ± s.e.

leukaemic cells were added beneath underlayers with optimal concentrations of HPCM and 5 different marrow non-adherent target cells (5 experiments) cultured in the overlayers. At lower cell concentrations (0·5–2 × $10^6$ leukaemic cells) there was a variable effect on normal marrow growth, 3/5 experiments showing increased growth. However, at a cell concentration of 8 × $10^6$, normal marrow growth was always inhibited (50–82% inhibition, mean 68%).

Suppression did not appear to affect any particular colony type, as results were equivalent when cultures were scored on Days 7 and 14. Examination of colony morphology on Day 14 did not reveal a differential suppression of eosinophil, granulocyte, macrophage, or granulocyte–macrophage colonies.

To further confirm that this inhibition was not unique to the leukaemic cells used in the initial experiments, further experiments were performed with other leukaemic-cell underlayers. Table I shows the results of 6 such experiments. In 2 experiments (Expts 1–4) inhibition was produced at only high cell numbers (8 × $10^6$) and in two other experiments (2 and 3) at lower cell numbers of (0·5 × $10^6$). Two cases of pure monocytic leukaemia, a known secretor of CSA, were also tested for colony inhibition. These cells did not suppress colony formation and, at high cell concentrations without HPCM, produced equivalent or more colonies than were produced with HPCM.

Inhibition at high concentrations in these experiments did not appear to be due to poor culture conditions secondary to nutrient depletion, as colony size was not affected. Colonies of several hundred cells each were regularly observed in cultures containing 8 × $10^6$ leukaemic cells, even though leukaemic growth frequently was not totally suppressed by the 0·5% agar overlayer.

As a further possible control against nutritional depletion of 8 × $10^6$ viable cells beneath the underlayer, up to 8 × $10^6$ normal peripheral blood cells were also cultured in underlayers with and without HPCM and with or without indomethacin. Any inhibition observed at high cell concentrations of normal cells was eliminated by the addition of indomethacin, suggesting a prostaglandin mechanism as described previously (Kurland *et al.*, 1978) and not a nutritional mechanism. It is interesting to note that mononuclear cells increased the stimulus over that observed with HPCM alone (Fig. 2). All 9 experi-

FIG. 2.—Influence of normal peripheral-blood cells with and without HPCM and indomethacin on colony formation of normal nonadherent marrow cells. Varying numbers of normal peripheral-blood mononuclear cells were cultured in agar underlayers with HPCM (△) and with $10^{-6}$M indomethacin (□) with both (■) and with neither (●). Bars represent s.e. based on 3 plates.

ments previously described that showed leukaemic inhibition of CFU-C growth were also performed incorporating $10^{-6}$M indomethacin and, unlike the results with normal cells, showed no abolition of marrow suppression. In some experiments there was enhancement of suppression at high cell doses, when indomethacin was included in cultures. One such experiment is shown in Table I. This is being further investigated, but it appears certain that prostaglandin synthesis and release are not the mechanism of inhibition.

To determine whether cell division was necessary for this suppression, leukaemic cells were irradiated before culture. There were no single cells or clusters in the underlayers when cultures were scored on Day 7 or 14. Results from these experiments are given in Fig. 3 (same leukaemic sample as Fig. 1) and in Table II for the leukaemics listed in Table I. In all cases in which irradiation was used, it diminished or abolished suppression, and enhanced colony growth significantly in some experiments. It did not destroy the ability of leukaemic cells to produce CSA (Expt 5, Table II).

If an inhibitor is produced by high concentrations of leukaemic cells, it should be possible to detect such an inhibitor by conditioning medium with leukaemic cells, providing it is not too labile. Media were conditioned for 1, 2, 3, or 7 days with varying concentrations of leukaemic cells (0·5–8 × $10^6$/ml) and 0·2 ml of media was placed in underlayers with HPCM. Effects of growth on nonadherent marrow targets were compared to those on cultures containing HPCM alone. In no instance was significant inhibition or stimulation of marrow growth detected (Table III, and other data not shown). No inhibitors or

TABLE II.—*Irradiated leukaemic-cell modulation of CFU-C growth*

| No. leukaemic cells (× $10^6$) | HPCM | Experiment 1 | 2 | 3 | 4 | 5† |
|---|---|---|---|---|---|---|
| 0 | + | 39 ± 2‡ | 112 | 33 ± 1 | 33 ± 1 | 39 ± 2 |
| | – | 0 | 0 | 0 | 0 | 0 |
| 0·5 | + | 75 ± 4 | 112 | 28 ± 1 | 53 ± 1 | 65 ± 5 |
| | – | 0 | 0 | 0 | 2 ± 1 | 13 ± 2 |
| 1·0 | + | 69 ± 2 | 104 | 15 ± 1 | 42 ± 3 | 65 ± 4 |
| | – | 0 | 4 | 0 | 5 ± 1 | 14 ± 1 |
| 2·0 | + | 72 ± 4 | 80 | 22 ± 5 | 46 ± 4 | 59 ± 1 |
| | – | 0 | 31 | 0 | 10 ± 2 | 62 ± 2 |
| 4·0 | + | 65 ± 4 | 49 | 28 ± 2 | 53 ± 3 | 51 ± 4 |
| | – | 0 | 46 | 0 | 26 ± 5 | 59 ± 5 |
| 8·0 | + | 63 ± 1 | 43 | 31 ± 2 | 51 ± 4 | 72 ± 3 |
| | – | 0 | 44 | 0 | 28 ± 4 | 51 ± 1 |

† Pure monocytic leukaemia.

FIG. 3.—Effect of irradiation of leukaemic cells on inhibition of normal colony growth. Normal marrow was cultured on leukaemic cell underlayers that either had (△, ▲) or had not (○, ●) been irradiated with 20 Gy before culture in the underlayer. (Solid symbols: Expt 1. Open symbols: Expt 2.)

TABLE III.—*Effect of leukaemic-conditioned media (Day 7) on normal marrow (non-adherent) CFU-C growth in response to CSA (No. of colonies (s.e.))*

| No. leukaemic cells to condition media (×10⁶) | Experiment 1 | 2 | 3 | 4 |
|---|---|---|---|---|
| 0 (control) | 12 (1) | 71 (4) | 105 (2) | 11 (1) |
| 0·5 | 11 (1) | — | — | — |
| 1 | 15 (1) | — | — | — |
| 2 | 11 (2) | 64 (0) | — | — |
| 4 | 9 (0·5) | — | — | — |
| 8 | 12 (1) | 79 (5) | 116 (2) | 16 (1) |

Media conditioned by varying concentrations of leukaemic cells for 7 days were added at a total volume of 0·2 ml to underlayers of 0·5% agar with optimal amounts of CSA (human placental-conditioned media). The control medium was α-MEM with 15% FCS and no cells

TABLE IV.—*Absorption of human placental-conditioned media (HPCM)*

| Source of cells | Expt 1 | Expt 2 |
|---|---|---|
| 0 | 17* (1)† | 12 (2) |
| Leukaemic A | 26 (2) | 24 (2) |
| Normal mononuclear blood cells | 28 (2) | 33 (2) |
| Normal low-density (< 1·077 g/ml) marrow cells | 19 (2) | 32 (1) |

* No. colonies.
† s.e.
HPCM was absorbed with $10^8$ cells/ml for 30 min at 4°C. A volume of 0·1 ml was then added to the underlayer and compared to unabsorbed HPCM using $10^5$ nonadherent, low-density (< 1·077 g/ml) human marrow cells as a target. Expts 1 and 2 represent different targets.

stimulators could be induced by including PHA in the culture while media were conditioned by leukaemic cells (data not shown). An alternative explanation could be that the high numbers of leukaemic cells consumed CSA, though this appeared unlikely in view of the normal colony size noted when inhibition was seen. To test this, we absorbed aliquots of HPCM with $10^8$ cells/ml for varying periods at 4°C or 37°C. Table IV shows the unexpected results. The HPCM absorbed with leukaemic cells, normal peripheral-blood mononuclear cells, or normal marrow cells caused higher colony growth than unabsorbed HPCM. This phenomenon was

TABLE V.—*Dose titrations of absorbed and unabsorbed HPCM*

| | Colony counts | | | |
|---|---|---|---|---|
| HPCM (ml) | Unabsorbed | Absorbed HPCM at 4°C for 60 min (A) | Absorbed HPCM at 4°C for 14 h | 0·1 ml unabsorbed HPCM + 0·2 ml absorbed (A) |
| 0·025 | 0 | 45 | 90 | — |
| 0·05 | 5 | 53 | 180 | — |
| 0·1 | 10 | 210 | 180 | — |
| 0·2 | 53 | 220 | 270 | — |
| 0·3 | 12 | 140 | 85 | 235 |

Varying amounts of absorbed or unabosrbed HPCM (except for one mixing experiment) were placed in agar underlayers and $10^5$ non-adherent, low-density (< 1·077 g/ml) marrow cells were used as a target in a 0·3% upper layer. Values are the mean of 2 plates.

not confined to this batch of HPCM, as similar results occurred with other batches. The enhancement was greater when absorption were continued for 60 min or overnight (Table V). Contrary to expectations, the possibility that an inhibitor of HPCM activity was absorbed was not borne out. Titrations with both absorbed and unabsorbed HPCM suggested that an inhibitor might be present in both preparations at the same volume of 0·3 ml, and mixing unabsorbed and absorbed HPCM did not suppress colony formation below that obtained with absorbed HPCM alone (Table V).

### DISCUSSION

These experiments show that leukaemic cells from some patients, when cultured in underlayers, can inhibit normal marrow myeloid colony growth. This inhibition occurs regularly only at high leukaemic cell concentrations (4–8 $\times 10^6$ cells culture) much greater than the concentrations reported to cause inhibition in co-culture experiments by other authors (Chiyoda *et al.*, 1975; Craddock *et al.*, 1978; Gordon *et al.*, 1978; Knudtson & Mortenson, 1976; Morris *et al.*, 1975). In fact, at low cell concentrations we frequently found stimulation of normal colony growth, despite the fact that these same leukaemic cell concentrations did not stimulate colony formation when used in underlayers without HPCM. A possible explanation for the difference between our observations and those of other reported co-culture experiments is that the culture conditions or CSA sources in the other studies were suboptimal. The other authors used lower criteria for colony size, which suggested poorer colony growth. In Chiyoda's work $2 \times 10^5$ cells gave $16{\cdot}4 \pm 2{\cdot}4$ colonies (mean $\pm$s.e.). This is about 10–25% of the figures obtained in our laboratory. Under suboptimal conditions, CFU-C may be more sensitive to leukaemic inhibitors. Other possible explanations for the discrepancy between the cell doses required include some intermediary inhibitor cell when feederlayers are used, or alternatively more short-range humoral inhibitors, only demonstrated when leukaemic cells are used in the overlayer.

It is unlikely that our results are due to poor culture conditions secondary to consumption of nutrients at higher cell concentrations. Colony size was not affected, a finding that has always been seen with poor culture conditions. Also, normal peripheral-blood mononuclear cells with indomethacin or pure monocytic leukaemic cells capable of releasing CSA did not suppress colony growth. Indomethacin did not abolish this leukaemic suppression, which rules out prostaglandin as the mechanism. This suppression probably required some cell division, for sterilization with radiation abolished it.

We expected that, if such suppression was demonstrable in agar culture, a soluble factor affecting this inhibition should be detected by allowing viable leukaemic cells in varying concentrations to condition media. This idea was supported by Broxmeyer's finding of a so-called luekaemic inhibitory activity (LIA), which can suppress CFU-C and can be detected in leukaemic extracts or leukaemic-conditioned media. Despite many attempts with cells from 4 leukaemic patients, including cells showing inhibition of CFU-C growth in co-culture experiments, we have not been able to detect such an inhibitor in conditioned media.

Bull *et al.* (1973) have suggested that leukaemic cells, in response to HLA-differing marrow, release inhibitory factors. Though mixtures of normal marrow do not show suppression (Chiyoda *et al.*, 1975; Morris *et al.*, 1975) this does not totally rule out the possibility. Also, using different target cells on the same leukaemic patient underlayers, suppression at high cell doses was within a narrow range, an unusual finding if HLA differences accounted for these results.

In the course of these investigations, other unexpected interactions in leukaemic and normal haematopoiesis were discovered. First, irradiation of leukaemic cells before their incorporation into agar culture frequently enhanced the growth of myeloid CFU-C over growth with HPCM alone. The HPCMs we have are routinely more active than human peripheral mononuclear cells, and probably represent the optimal reproducible system for growth of human myeloid CFU-C (Verma *et al.*, 1980). Secondly, absorption of HPCM at 4°C improved HPCM activity markedly over that at 37°C. This was not due to removal of inhibitor, and the improvement in activity was not specific to leukaemic absorption. It is conceivable that HPCM could interact with the cell membrane, be altered in biochemical composition, and then come off the cell membrane in a more active form. Absorption at 37°C might be associated with production of proteases, and breakdown of this more active moiety. Irradiated cells in culture may alter HPCM in a similar way, enhancing cell growth. Metabolism may not be necessary for this membrane interaction. Unirradiated leukaemic cells could show variable effects, depending upon secretion of CSA or possible inhibitors, possible favourable alterations in the CSA molecule, and the release of enzymes that could break down this favourably altered CSA. Because of the favourable effects of leukaemic addition to agar culture, inhibition may not be detected until enough leukaemic cells are added to secrete significant amounts of inhibitors. Obviously, the interactions of leukaemic cells with CFU-C are far from understood and require much more investigation. Lastly, it may not even occur at the level of the myeloid CFU-C, but at earlier stages in haemopoiesis. These results may therefore merely represent interesting *in vitro* findings.

This work was supported in part by the National Cancer Institute, Grants Number CA-11520 and CA-14525.

G. Spitzer is a Scholar of the Leukemia Society of America, Inc.

### REFERENCES

ADAMSON, J. W. (1979) Analysis of myeloproliferative disorders using cell markers in cultures. In *Modern Trends in Human Leukemia*. Eds Heth *et al.* Berlin: Springer-Verlag. p. 250.

BROXMEYER, H. E., GROSSBARD, E., JACOBSEN, N. & MOORE, M. A. S. (1978*a*) Evidence for a proliferative advantage of human leukemic colony forming cells *in vitro*. *J. Natl Cancer Inst.*, **513**, 521.

BROXMEYER, H. E., JACOBSEN, N., KURLAND, J., MENDELSOHN, N. & MOORE, M. A. S. (1978*b*) *In vitro* suppression of normal granulocytic stem cells by inhibitory activity derived from human leukemic cells. *J. Natl Cancer Inst.*, **497**, 511.

BROXMEYER, H. E., GROSSBAND, E., JACOBSEN, N. & MOORE, M. A. S. (1979) Persistence of inhibitory activity against normal bone marrow cells during remission of acute leukemia. *N. Engl. J. Med.*, **346**, 351.

BULL, J. M., ROSENBLUM, A. L. & CARBONE, P. P. (1973) *In vitro* culture studies in patients with acute myelocytic leukemia. In *Second International Workshop on Hematopoiesis in Culture*. Ed Robinson. Washington, D.C.: HEW Publications No. (NIH) 74205. p. 335.

CHIYODA, S., MIZOGUCHI, H., ASANO, S., TAKAKU, F. & MURIA, Y. (1976) Influence of leukaemic cells on the colony formation of human bone marrow cells *in vitro*, II. Suppressive effects of leukaemic cell extracts. *Br. J. Cancer*, **33**, 379.

CHIYODA, S., MIZOGUCHI, H., KOSAKA, K., TAKAKU, F. & MIURA, Y. (1975) Influence of leukemic cells on the colony formation of human bone marrow cells *in vitro*. *Br. J. Cancer*, **31**, 355.

CRADDOCK, C. G., RODENSKY, O., FORSEN, N. R., KOSPERSKY, C. J. & SPARKES, R. S. (1978) Myeloblastic leukemic cells causing suppression of granulopoiesis and excessive binding of colony stimulating activity. *Am. J. Med.*, **64**, 343.

FIAKLOW, P. J., SINGER, J. W., ADAMSON, J. W. & 4 others (1979) Acute nonlymphocyte leukemia: Expression in cells restricted to granulocytic and monocytic differentiation. *N. Engl. J. Med.*, **301**, 1.

GORDON, N. Y., DOUGLAS, F. D. C. & BLACKETT, N. M. (1978) Effect of medium conditioned by acute myeloid leukaemia cells on normal marrow colony forming ability and sensitivity to cytosine arabinoside. *Eur. J. Cancer*, **14**, 661.

GREENBERG, P. L., NICHOLS, W. C. & SCHRIER, S. L. (1971) Granulopoiesis in acute myeloid leukemia and preleukemia. *N. Engl. J. Med.*, **284**, 1225.

KNUDTSON, S. & MORTENSEN, B. T. (1976) Interaction between normal and leukaemic human cells in agar culture. *Scand. J. Haematol.*, **17**, 369.

KURLAND, J. J., BROXMEYER, H. E., PELUS, L. M., BOCKMAN, R. S. & MOORE, M. A. S. (1978) Role for monocyte–macrophage derived colony stimulating factor and prostaglandin E in the positive and negative feedback control of myeloid stem cell proliferation. *Blood*, **52**, 388.

MOORE, M. A. S. & METCALF, D. (1973) Cytogenetic analysis of human acute and chronic myeloid leukemic cells cloned in agar culture. *Int. J. Cancer*, **11**, 143.

MOORE, M. A. S., WILLIAMS, N. & METCALF, D. (1973) *In vitro* colony formations by normal and leukemic human hematopoietic cells. *J. Natl Cancer Inst.*, **50**, 603.

MOORE, M. A. S., SPITZER, G., WILLIAMS, N., METCALF, D. & BUCKLEY, J. (1974) Agar culture studies in 127 cases of untreated acute leukemia: The prognostic value of reclassification of leukemia according to *in vitro* growth characteristics. *Blood*, **44**, 1.

Morris, J. C. M., McNeill, T. A. & Bridges, J. M. (1975) Inhibition of normal human *in vitro* colony-forming cells by cells from leukaemic patients. *Br. J. Cancer*, **31**, 641.

Robinson, W. A., Kurnick, J. E. & Pike, B. L. (1971) Colony growth of human leukemic peripheral blood *in vitro*. *Blood*, **38**, 500.

Senn, J. S., McCulloch, E. A. & Till, J. E. (1967) Comparison of colony-forming ability of normal and leukaemic human marrow in cell culture. *Lancet*, ii, 597.

Spitzer, G., Dicke, K. A., Gehan, E. A. & 4 others (1976*a*) A simplified *in vitro* classification for prognosis in adult acute leukemia: The application of *in vitro* results in remission predictive models. *Blood*, **48**, 795.

Spitzer, G., Dicke, K. A., Gehan, E. A., Smith, T. & McCredie, K. B. (1976*b*) The use of the Robinson *in vitro* agar culture assay in adult acute leukemia. *Blood Cells*, **2**, 139.

Spitzer, G., Dicke, K. A., McCredie, K. B. & Barlogie, B. (1977) The early detection of remission in acute myelogenous leukaemia by *in vitro* agar culture. *Br. J. Haematol.*, **35**, 411.

Spitzer, G., Verma, D. S., Barlogie, B., Beran, M. A. & Dicke, K. A. (1979) Possible mechanisms of action of lithium on augmentation of *in vitro* spontaneous myeloid colony formation. *Cancer Res.*, **39**, 3215.

Verma, D. S., Spitzer, G., Beran, M., Zander, A. R., McCredie, K. B. & Dicke, K. A. (1980) Colony stimulating factor augmentation in human placental conditioned medium. *Exp. Hematol.*, **8**, 917.